# High avoidance and low approach motivation affect cognitive reappraisal generation in the face of anger

**DOI:** 10.1007/s12144-021-01917-y

**Published:** 2021-05-31

**Authors:** Corinna M. Perchtold-Stefan, Andreas Fink, Jonathan F. Bauer, Christian Rominger, Ilona Papousek

**Affiliations:** Department of Psychology, University of Graz, Universitätsplatz 2, 8010 Graz, Austria

**Keywords:** Reinforcement sensitivity, Cognitive reappraisal, Approach, Avoidance, Emotion regulation

## Abstract

This study investigates the link between the revised reinforcement sensitivity theory (RST) and individuals’ capacity to spontaneously invent alternate appraisals for aversive events. Eighty-two women completed the Reappraisal Inventiveness Test for anger-eliciting situations, and quantity and quality of reappraisal ideas were correlated with BIS, BAS, and FFFS sensitivity (RST-Personality Questionnaire). Results revealed that high BIS and high FFFS reduced the total number of reappraisal ideas, indicating that combined heightened sensitivity of the two avoidance systems may deplete individuals’ repertoire of potential reappraisals. RST effects on the quality of generated reappraisals were also found. High BIS and low BAS lowered individuals’ propensity to produce positive re-interpretations, which are considered a more adaptive reappraisal tactic. High FFFS sensitivity was linked to a lower preference for problem-oriented reappraisals. This study underlines that certain interactions of motivational subsystems may be particularly detrimental for successful reappraisal of anger-eliciting events. Our findings reveal potential links between revised RST effects and adaptive emotion regulation.

## Introduction

The idea that neurobehavioral systems initiating appetitive and aversive motivation may also exert critical influence on the regulation of affective processes has gained substantial attention in recent years (e.g., Corr, 2013; Markarian et al., 2013; Tull et al., 2010). A rather influential concept in this regard is the reinforcement sensitivity theory of personality (RST), which in its revised version postulates three major brain subsystems as key modulators of human emotion (Gray & McNaughton, 2000; McNaughton & Corr, 2004, 2008): The behavioral approach system (BAS), the behavioral inhibition system (BIS), and the Fight-Flight-Freeze System (FFFS). According to the revised RST, the BAS coordinates approach behavior and is responsive to reward and appetitive stimuli, while the FFFS is a defensive avoidance system activated by aversive stimuli and linked to fear. The BIS is considered to be involved in resolution of goal conflict by inhibiting behavior and facilitating risk assessment for e.g., approach-avoidance conflicts, which elicit anxiety (for an extensive discussion see Corr, 2008). The distinction between the BIS and the FFFS in the revised RST presents the most significant change from the original RST postulated by Gray (1982), where only BAS and BIS were emphasized. While there is ample evidence linking the original RST to individual differences in emotion regulation (difficulties) and by extension, psychopathology like anxiety, mood, or personality disorders (e.g., Bijttebier et al., 2009; Hundt et al., 2013; Tull et al., 2010), it is still relatively unknown how the reconceptualization of the RST relates to models of emotion regulation. This is particularly interesting as regards specific, presumably more adaptive emotion regulation strategies like cognitive reappraisal, which even in the original RST framework, have largely been neglected due to a strong focus on maladaptive emotion regulation in the more general sense (also see Hamill et al., 2015). For this reason, the present study investigated the relationship between BIS/BAS/FFFS sensitivity of the revised RST and individuals’ capability to ad hoc generate manifold cognitive reappraisals for negative emotional events, which is called reappraisal inventiveness (Weber et al., 2014).

### Reinforcement Sensitivity Theory and Emotion Regulation

Emotion regulation is considered a central mechanism through which BIS-FFFS^1^ and BAS sensitivity influence psychopathology, but also psychological well-being (e.g., Azadi et al., 2020; Bijttebier et al., 2009; Harnett et al., 2013; Izadpanah et al., 2016; Tull et al., 2010). The majority of literature demonstrates robust links between higher BIS-FFFS sensitivity and general emotion regulation difficulties as well as sustained negative affect (e.g., Hannan & Orcutt, 2013; Hundt et al., 2007, 2013; Markarian et al., 2013; Tull et al., 2010), with a few studies underlining this relationship for specific maladaptive strategies like avoidance (Pickett et al., 2012) or angry rumination (Izadpanah et al., 2017). Longitudinal investigations substantiate that BIS-FFFS sensitivity and maladaptive emotion regulation interact to increase vulnerability towards anxiety and depression (e.g., Izadpanah et al., 2016; Li et al., 2015). In this regard, Izadpanah et al. (2016) showed that BIS-FFFS sensitivity predicted maladaptive emotion regulation and anxiety years later, while Li et al. (2015) established similar effects on depressive symptoms. While higher BIS-FFFS sensitivity seems to underlie general maladaptive responses to emotions, for BAS sensitivity, this relationship is more complex. Despite being related to more positive affect (e.g., Hundt et al., 2013), BAS sensitivity has previously shown only weak and rather inconsistent associations with emotion regulation. Markarian et al. (2013) found low BAS sensitivity indirectly linked to depression through emotion regulation difficulties, whereas Serrano-Ibáñez et al. (2018) reported BAS sensitivity to be unrelated to both adaptive and maladaptive emotion regulation. Moreover, directions of effects sometimes only emerge for specific dimensions of BAS (e.g., BAS-reward responsiveness, BAS-drive; Markarian et al., 2013; Tull et al., 2010; also see Taubitz et al., 2015). Importantly, the joint subsystems hypothesis of the RST (Corr, 2002) postulates that BIS-FFFS and BAS effects may not be independent, but in fact interact in their influence on emotional behavior. In this regard, Markarian et al. (2013) suggested that joint effects of high BIS-FFFS and low BAS may lead to particular emotion regulation difficulties. Similarly, interactions between BIS-FFFS and BAS have been linked with unfavorable mental health impacts in terms of depression and anxiety (e.g., O’Connor et al., 2014; Harnett et al., 2013; Hundt et al., 2007). Interestingly, there is also evidence that BAS sensitivity may be more strongly linked to the use of adaptive emotion regulation strategies than the use of maladaptive ones (e.g., Azadi et al., 2020; Izadpanah et al., 2016). Together with the fact that reinforcement sensitivity research is lacking detailed investigations of both *specific* and *adaptive* emotion regulation strategies, this warrants a closer investigation of BIS/BAS/FFFS sensitivity in relation to adaptive emotion regulation strategies like cognitive reappraisal.

### Reinforcement Sensitivity Theory and Cognitive Reappraisal

Cognitive reappraisal is believed to alter the impact of emotional events by purposefully viewing them from different, perhaps even novel perspectives (e.g., Gross & John, 2003, also see Weber et al., 2014). This re-interpretation of the personal significance of emotional situations is broadly considered very powerful in dealing with life’s adversity (e.g., Webb et al., 2012, also see Liu et al., 2019). Among the small number of studies linking reinforcement sensitivity to specific adaptive emotion regulation strategies (e.g., mindfulness: Hamill et al., 2015; problem-focused coping: Hundt et al., 2013), some have specifically scrutinized links with cognitive reappraisal. O’Connor et al. (2014) found lower use of cognitive reappraisal in individuals high in threat sensitivity, a finding that was later supplemented by Serrano-Ibáñez et al. (2018), who likewise asserted strong negative links between BIS-FFFS sensitivity and self-reported cognitive reappraisal use. In both studies, cognitive reappraisal acted as a mediator between BIS-FFFS and negative affect. Conversely, higher BAS sensitivity in terms of reward responsiveness was significantly correlated with increased self-reported use of cognitive reappraisal (Cooper et al., 2008; Taubitz et al., 2015). While it may thus seem intuitive that cognitive reappraisal as an adaptive emotion regulation strategy is negatively correlated with BIS-FFFS and positively correlated with BAS, there are also discrepant reports (see Dennis, 2007), which requires clarification. Overall, the small number of RST investigations of cognitive reappraisal is surprising, considering that cognitive reappraisal is often exemplified as approach-oriented coping technique that necessitates approaching the emotional content (e.g., Folkman & Moskowitz, 2000; Llewellyn et al., 2013; Wiley et al., 2016), and effective use of cognitive reappraisal is markedly reduced in populations high in experiential avoidance (e.g., social anxiety; Aldao et al., 2010; Dryman & Heimberg, 2018; Goldin et al., 2009). Accordingly, the present study aimed to provide further information on the relationship between cognitive reappraisal and BIS/BAS/FFFS sensitivity, by using a specific index of cognitive reappraisal: individuals’ inventiveness in generating alternate appraisals for self-relevant, aversive scenarios.

### Reappraisal Inventiveness: Quantity and Quality of Reappraisals

A different approach to assessing individuals’ self-reported tendencies for using cognitive reappraisal, reappraisal inventiveness denotes individuals’ capacity to spontaneously generate manifold alternative appraisals for emotionally-evocative situations (Weber et al., 2014). The Reappraisal Inventiveness Test (RIT; Weber et al., 2014) asks participants to generate multiple reappraisals for anger-eliciting situations that may reduce their anger. Objective, rater-based analysis of the generated reappraisal ideas in terms of quantity and appropriateness then results in an index of overall inventiveness (number of ideas). Past research has linked reappraisal inventiveness in the RIT to more adequate activation of the left lateral prefrontal cortex during reappraisal generation (Papousek et al., 2017; Perchtold et al., 2019), with this brain activation pattern also predicting indices of emotional functioning in daily life, like chronic stress perception and hostile attributions (Perchtold et al., 2018, 2019). This suggests that the capacity to invent manifold different interpretations for emotional events may qualify as a brain-based construction competence, which may serve as a direct foundation for effective reappraisal implementation in everyday contexts (Papousek et al., 2017; Weber et al., 2014). Since the RST is a neurobiologically-based theory of personality (Gray & McNaughton, 2000), it is particularly interesting to examine how its revised conceptualizations of BIS/BAS/FFFS are linked to cognitive reappraisal generation when it is operationalized as a brain-based capacity. Additionally, it is crucial to note that cognitive reappraisal is also comprised of a variety of different tactics or qualities of ideas, which may entail very different cognitive reframing of emotional scenarios (e.g., McRae et al., 2012; Shiota & Levenson, 2009). Next to scoring the commonly distinguished reappraisal tactics of positive (situation-focused) reappraisal and detached (de-emphasizing) reappraisal (e.g., Ochsner et al., 2012; Shiota & Levenson, 2012), the RIT also assesses the number of problem-oriented reappraisals geared towards action-based solutions for emotional events (Weber et al., 2014). Assessing the specific content of reappraisal ideas generated in the RIT may provide critical insights into what types of reappraisals individuals preferably implement in daily life. Importantly, given that different reappraisal tactics are linked with different health outcomes (Kalisch et al., 2015; Perchtold et al., 2018, Perchtold-Stefan et al., 2020), it may provide vital information to determine how individual differences in BIS/BAS/FFFS sensitivity influence individuals’ preference for certain reappraisal strategies over others.

### The Present Study

While there is some evidence that high BIS-FFFS and low BAS may impair the self-reported use of cognitive reappraisal (e.g., O’Connor et al., 2014; Serrano-Ibáñez et al., 2018; Taubitz et al., 2015), it remains unclear how these neurobiological systems according to the revised RST influence cognitive reappraisal generation when measured as reappraisal inventiveness. For this reason, the present study investigated the relationships between BIS, BAS, and FFFS sensitivity and quantity and quality of reappraisal ideas in the face of anger-eliciting events (Weber et al., 2014). We purposely focused on cognitive reappraisal of anger, since anger has received considerably less attention in the RST framework than anxiety or depression, despite being linked to both BIS-FFFS and BAS sensitivity (Carver, 2004; Cooper et al., 2008; Smits & Kuppens, 2005; Tibubos et al., 2014). Further, given that anger is linked to approach motivation (see Harmon-Jones, 2003), it may also help clarify the hitherto inconsistent role of BAS sensitivity in adaptive emotion regulation. In our multiple regression models, we considered both main effects of BIS, BAS, and FFFS as well as interaction effects of these systems on reappraisal parameters, given that the joint subsystems hypothesis regards behavior as the result of multiple state systems being activated (Corr, 2002). Since research assumes a joint contribution of BIS and FFFS to aversive motivational processes (Corr, 2008, also see O’Connor et al., 2014) and no prior research has disentangled effects of BIS and FFFS on cognitive reappraisal, we assumed that high sensitivity to both systems would reduce overall reappraisal inventiveness (i.e., quantity of generated ideas). This relationship may either manifest in two main effects of BIS and FFFS or their statistical interaction (see Corr, 2013). We did not formulate strong a priori predictions concerning links between BIS/BAS/FFFS and use of specific reappraisal tactics (i.e., reappraisal quality); however, we expected that high BAS sensitivity may be linked to a greater preference for positive re-interpretations, since both incorporate aspects of reward and are related to positive affect (e.g., Hundt et al., 2013; Shiota & Levenson, 2012).

## Methods

### Participants

The final sample consisted of *N* = 82 female university students, with a mean age of *M* = 23.88 (*SD* = 4.22; age range between 18 and 37 years). G*Power was used to estimate the required sample size a priori (α = .05, 1 − β = 0.80), which yielded a minimum of 75 participants for a multiple regression approach. Calculations were run with an effect size of f^2^ = .20, which we deemed a good estimate based on varying effect sizes in previous relevant research (f^2^ = .06 to .33; O’Connor et al., 2014; Serrano-Ibáñez et al., 2018, also see Llewellyn et al., 2013). Recruiting took place online via social media channels and via university mail, and offline via posters placed on various university campuses. Interested individuals were phoned to check for exclusion criteria (no intake of drugs or psychoactive medication, no neurological/psychiatric history) and to arrange an appointment. Initially, 85 participants signed up for study participation; however, three individuals did not show up at their designated appointment. No participant was excluded after testing. Written consent for study participation was obtained from all participants, who completed the questionnaire on reinforcement sensitivity (RST-PQ) before the Reappraisal Inventiveness Test (RIT). The study was approved by the authorized local Ethics Committee.

### Reappraisal Inventiveness Test (RIT)

In the RIT (Weber et al., 2014), participants are confronted with self-relevant, anger-eliciting situations that match common, everyday experiences where harmful behavior by another person occurs deliberately or carelessly. In the kitchen item of the RIT, for example, participants are presented with the scenario that they arrive at their flat and find their kitchen a mess, although their roommate promised to clean it before guests arrive this evening (see Weber et al., 2014, p. 360). These items are designed to be relatable, easily imaginable, and at most, moderately anger-evoking (e.g., Sheppes et al., 2014) in order to prompt the generation of manifold reappraisal ideas for the downregulation of the concomitant negative emotions. In the experimental setting of this study, each of the RIT vignettes (six in total) were presented on a computer screen for 20 s, together with a matching photograph to increase immersion. Participants were instructed to picture the situation as vividly as possible in their minds, and subsequently generate as many different ideas as possible to reappraise the scenario in a way that reduced their experienced anger. This ideation phase lasted for 3 min, with participants pressing a button whenever an idea occurred to them and vocalizing it into a microphone, which audiotaped all ideas for later analysis. This procedure was explained with a practice item in order to familiarize participants with the task requirements. At the end of the task, participants rated the anger they would experience when facing these scenarios in real-life (7-point scales ranging from 0 “not angry at all” to 6 “very angry”; *M* = 3.55, *SD* = 0.75). Ratings indicated that all items were perceived as potentially anger-evoking. Total duration of the RIT was ~20 min.

Two experienced researchers independently calculated the total number (fluency) of non-identical reappraisals generated for all RIT items, which was then averaged into a total reappraisal inventiveness score due to satisfying intraclass correlations (ICC, 95% confidence intervals, consistency) of .99 (for a similar procedure and resulting ICCs, see Fink et al., 2017; Papousek et al., 2017; Perchtold et al., 2019; Weber et al., 2014). On average, participants generated *M* = 35.80 reappraisal ideas (*SD* = 13.74; *Min* = 5, *Max* = 64). In order to determine reappraisal quality, reappraisal ideas were coded by content according to the category scheme of the RIT (Weber et al., 2014). These three main, most commonly implemented reappraisal tactics are: problem orientation (action planning and finding ways to reduce harm), positive re-interpretation (perspective change in terms of generating positive aspects), and de-emphasizing (distancing and trivializing the meaning of a scenario). Reappraisal ideas from other, rarely used categories were excluded from the analyses (also see Perchtold-Stefan et al., 2020). Inter-rater reliabilities were ICC = .97, ICC = .96, and ICC = .98, for problem-oriented reappraisals, positive re-interpretations, and de-emphasizing reappraisals, respectively. Indices for individuals’ relative preference for implementing a certain reappraisal tactic were computed by dividing the number of reappraisal ideas from eligible categories (i.e., total number of problem-oriented reappraisals) by total ideational fluency, which yielded percentage scores for each reappraisal tactic. On average, 34.45% of reappraisal ideas qualified as problem-oriented (*SD* = 21.43, *Min* = 0, *Max* = 88.89), 18.26% of ideas were positive re-interpretations (*SD* = 12.67, *Min* = 0, *Max* = 61.54), and 37.66% of ideas were de-emphasizing reappraisals (*SD* = 24.40, *Min* = 0, *Max* = 100).

### Reinforcement Sensitivity

The Reinforcement Sensitivity Theory Personality Questionnaire (RST-PQ, Corr & Cooper, 2016) consists of 65 items measuring three major motivational systems: The Fight/Flight/Freeze System (FFFS,^2^ 10 items; e.g., “There are some things I simply cannot go near”), the Behavioral Inhibition System (BIS, 23 items; e.g. “I take a long time to make decisions”), and the *Behavioural Approach System* (BAS, 32 items). In the RST-PQ, BAS is defined as a multidimensional construct comprised of *Reward Interest* (7 items, e.g., “I am very open to new experiences in life”), *Goal-Drive Persistence* (7 items, e.g., “I often overcome hurdles to achieve my ambitions”), *Reward Reactivity* (10 items, e.g., “I am especially sensitive to reward”), and *Impulsivity* (8 items, e.g., “If I see something I want, I act straight away”). Participants answer on a scale from 1 (not at all) to 5 (highly) how well each statement describes them. Descriptive statistics of the RST-PQ are reported in Table 1.

**Table 1:** Descriptive statistics for the RST-PQ

	*M*	*SD*	*Min*	*Max*	*α*
Flight/Freeze/Avoidance (FFFS)	2.44	0.60	1.33	3.93	.77
Behavioral Inhibition (BIS)	2.32	0.52	1.04	3.44	.90
Behavioral Activation (BAS)	2.90	0.37	1.95	3.75	.84
*Reward Interest*	2.95	0.65	1.57	4.00	.85
*Reward Reactivity*	3.05	0.41	2.20	4.00	.68
*Goal-Drive Persistence*	3.05	0.62	1.85	4.00	.85
*Impulsivity*	2.46	0.41	1.25	3.25	.67

M = mean, SD = standard deviation, Min = Minimum, Max = Maximum, α = Cronbach Alpha

### Statistical Analysis

Four multiple regression analyses were run for testing the main research questions, one with total reappraisal inventiveness (fluency) and three with reappraisal quality as in the relative use of one of the three reappraisal categories of problem-solving, positive re-interpretation, and de-emphasizing. The three motivational systems FFFS, BIS, and BAS and the two-way interaction terms between FFFS, BIS, and BAS were added simultaneously, based on the assumption that these systems also represent a dynamically interacting network in which the behavioral output of each system is determined by an interaction between them (Corr, 2002, 2013). We chose this statistical approach since it allowed us to examine whether individual differences in FFFS, BIS, and BAS as well as their interactions explained a significant amount of variance in different cognitive reappraisal measures beyond the variance explained by other dimensions and their interactions. In this first investigation of the revised RST and reappraisal inventiveness, we were primarily interested in significant regression weights of single predictors, that is, specific effects of the RST dimension or specific interactions on indicators of reappraisal inventiveness. For this reason, significant effects of single predictors were interpreted even if the regression models were not significant. Significant interaction terms were further followed up with the SPSS macro PROCESS set to Model 1 (Hayes & Preacher, 2014), using simple slope analyses to illustrate the interaction effect across different levels of the moderator (1 SD below/mean/1 SD above mean). Based on previous studies denoting the diversity of the BAS scales in the RST-PQ (Corr & Cooper, 2016; Krupić et al., 2016), significant main and interaction effects with BAS were followed up by running the regression model for each of the four BAS scales, in order to determine whether correlations with indices of reappraisal were driven by a specific aspect of the BAS (reward reactivity, reward interest, impulsivity, goal-drive persistence). The statistical assumptions for the model were met (i.e., linearity, homoscedasticity, normality, independence of errors, ratio of cases to independent variables, and absence of multicollinearity). As supplementary analyses, regression models were computed with an additional three-way interaction term of BIS, BAS, and FFFS. In addition, intercorrelations between the RST-PQ scales were calculated. A significance level of *p* < .05 (two-tailed) was applied for all analyses.

## Results

### Reinforcement Sensitivity and Total Reappraisal Inventiveness (Reappraisal Quantity)

**Fig. 1:**
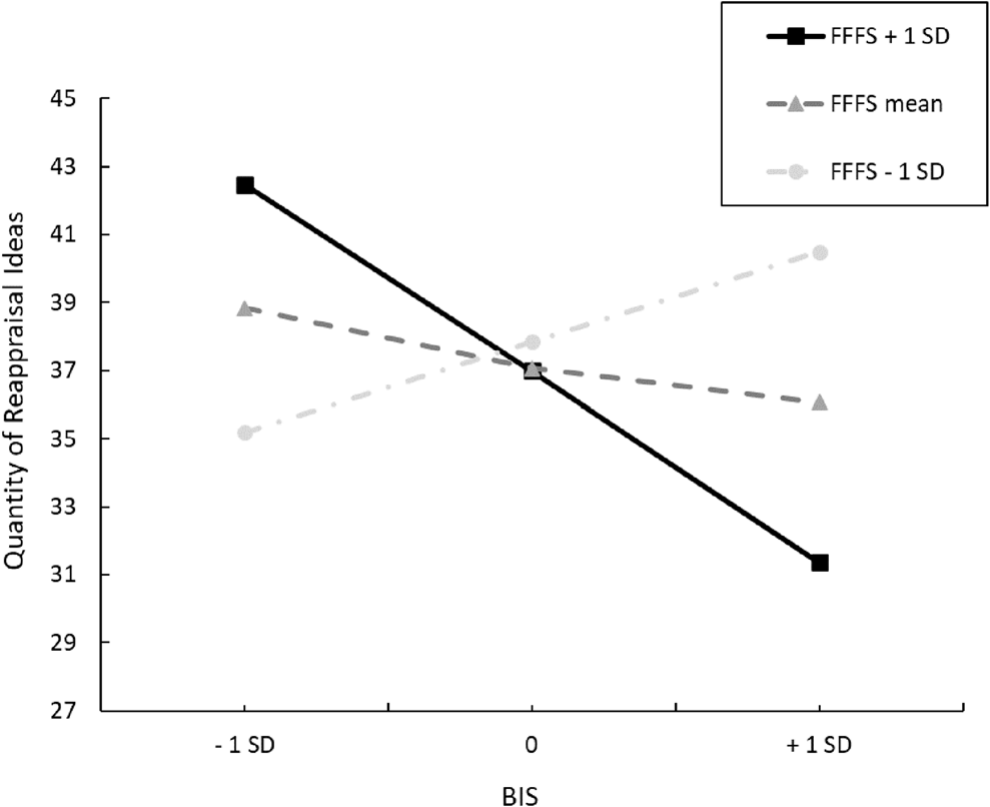
Interaction effect for BIS by FFFS on total reappraisal inventiveness. Note. BIS is linked to quantity of generated reappraisals only at high levels of FFFS (+ 1 SD; black line)

Neither the BAS, nor the BIS or FFFS dimensions were correlated with total reappraisal inventiveness. Among the interactions between motivational systems however, the interaction of BIS x FFFS was significant (*β* = −.24, *p* = .040; *F*(6,75) = 1.37, *p* = .240; see Table 2). Simple slope analyses revealed that BIS was related to the quantity of generated reappraisals only at higher (+1 SD) levels of FFFS (*p* = .017), denoting that participants high in BIS and high in FFFS generated the lowest number of reappraisal ideas (see Fig. 1).

**Table 2:** Summary of hierarchical multiple regression results for the total quantity and specific quality of generated reappraisal ideas

	Total quantity of reappraisals		Share of problem-oriented reappraisals		Share of positive re-interpretations		Share of de-emphasizing reappraisals	
	*β (p)*	*R*^*2*^ *(p)*	*β (p)*	*R*^*2*^ *(p)*	*β (p)*	*R*^*2*^ *(p)*	*β (p)*	*R*^*2*^ *(p)*
BAS	.06 (.647)	.10 (.240)	.13 (.287)	.07 (.434)	.06 (.616)	.11 (.199)	−.16 (.205)	.07 (.508)
BIS	−.12 (.313)		.10 (.412)		−.15 (.216)		−.03 (.854)	
FFFS	.01 (929)		**−.25 (.038)**		.15 (.209)		.21 (.078)	
BAS x FFFS	−.02 (.886)		.03 (.823)		−.11 (.376)		.01 (.970)	
BIS x FFFS	**−.24 (.040)**		−.03 (.827)		−.13 (.278)		.04 (.759)	
BIS x BAS	.15 (.208)		−.04 (.726)		**.25 (.036)**		−.02 (.880)	

Significant correlations are highlighted in bold font. Abbreviations: β = standardized beta weights; R^2^ = proportions of variance explained by the model

### Reinforcement Sensitivity and Quality of Generated Reappraisals (Reappraisal Tactics)

#### Problem-Oriented Reappraisal

FFFS was negatively correlated with the share of problem-oriented reappraisals (*β* = −.24, *p* = .037). However, none of the interactions between motivational systems explained unique variance in the share of problem-oriented reappraisals (*F*(6,75) = 1.00, *p* = .434; see Table 2 for details).

#### Positive Re-interpretation

**Fig. 2:**
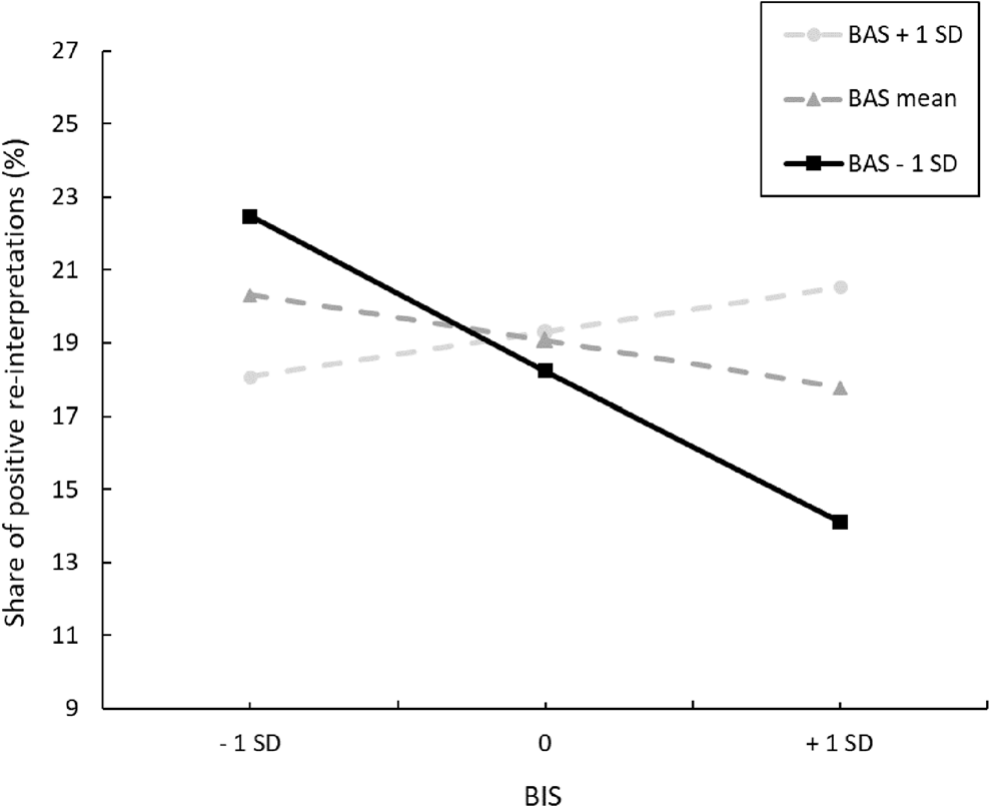
Interaction effect for BIS by BAS on share of positive re-interpretations. Note. BIS is linked to the share of positive re-interpretations only at low levels of BAS (−1 SD; black line). This BIS x BAS interaction effect is presented for the BAS scales of Reward Interest and Impulsivity, but not Reward Reactivity or Goal-Drive-Persistence

Neither the FFFS, nor the BIS or BAS dimensions were correlated with the share of positive re-interpretations. Among the interaction terms however, the contribution of BIS x BAS was significant (*β* = .25, *p* = .036; *F*(6,75) = 1.47, *p* = .199; see Table 2). Simple slope analyses revealed that BIS was associated with the share of positive re-interpretations only at lower (−1 SD) levels of BAS (*p* = .041), indicating that participants with high BIS and low BAS generated the lowest share of positive re-interpretations (see Fig. 2). Additional analyses revealed that this interaction effect was present for the BAS scales of reward interest (*β* = .27, *p* = .023) and impulsivity (*β* = .29, *p* = .013), but not for reward reactivity (*β* = .11, *p* = .363), and goal-drive persistence (*β* = .07, *p* = .568).

#### De-emphasizing

Neither the FFFS, nor the BIS or BAS dimensions were correlated with the share of de-emphasizing reappraisals. Additionally, none of the interactions between motivational systems were significant (*F*(6,75) = 0.89, *p* = .508; see Table 2).

### Supplementary Analyses

The three-way interaction of BIS, BAS, and FFFS had no significant effects on either quantity or quality of reappraisals in the RIT (all *p* values > .323) but left the previous pattern of results virtually unchanged. BAS was correlated with BIS (*r* = −.29, *p* = .008) but uncorrelated with FFFS (*r* = −.07, *p* = .524). BIS and FFFS were correlated at *r* = .27 (*p* = .015). For intercorrelations with the four BAS scales, see Table 3.

**Table 3:** Intercorrelations between RST-PQ scales

	BIS	FFFS	BAS	*BAS RI*	*BAS GDP*	*BAS RR*	*BAS IM*
BIS	1						
FFFS	.27^*^	1					
BAS	−.29^**^	−.07	1				
*BAS RI*	−.41^**^	−.32^**^	.71^**^	1			
*BAS GDP*	−.17	.03	.78^**^	.27^*^	1		
*BAS RR*	−.04	.19	.59^**^	.32^**^	.26^*^	1	
*BAS IM*	−.01	−.04	.34^**^	.29^**^	−.12	.37^**^	1

** *p* < .01, * *p* < .05. Abbreviations: *RI =* Reward Interest, *GDP* = Goal-Drive Persistence, *RR* = Reward Reactivity; *IM* = Impulsivity

## Discussion

The present study investigated the relationship between BIS, BAS, and FFFS sensitivity according to the revised RST and reappraisal inventiveness assessed in a behavioral performance test. In general, our results indicate that all three motivational subsystems influence individuals’ generation of cognitive reappraisals for anger-eliciting events; however, specific subsystems seem to affect specific aspects of reappraisal.

For overall reappraisal inventiveness, we observed an interaction of BIS and FFFS, demonstrating that the two avoidance systems interact to reduce the quantity of generated reappraisal ideas. Analyses revealed that high BIS sensitivity only affected reappraisal inventiveness at high levels of FFFS, with respective individuals generating the lowest number of valid reappraisal ideas. Although this study is the first to disentangle effects of BIS and FFFS on a brain-based capacity measure of cognitive reappraisal, the obtained results can be well integrated into existing literature.

First, BIS-FFFS sensitivity is generally linked to lower self-reported use of cognitive reappraisal (e.g., O’Connor et al., 2014; Serrano-Ibáñez et al., 2018; Taubitz et al., 2015), also in the specific case of regulating anger (Cooper et al., 2008). Seeing that these studies did not distinguish BIS and FFFS, their results may be indicative of combined effects of BIS and FFFS on impairing cognitive reappraisal generation. Arguing from the perspective of the revised RST may offer a more refined interpretation however. In the revised RST, activation of the BIS is supposed to inhibit predominant conflicting behaviors in the presence of approach-avoidance conflicts, which leads to ruminative cognition, increased risk assessment, and behavioral disengagement (e.g., Corr & Cooper, 2016). Generating manifold reappraisals for resolving anger-eliciting social situations likely elicits approach-avoidance conflicts, since anger is broadly linked to approach motivation (e.g., Carver & Harmon-Jones, 2009; Harmon-Jones, 2003), yet at the same time, individuals may also seek to avoid the depicted aversive confrontations with wrongdoers in favor of disengaging from the problem altogether. Given that cognitive reappraisal generation in the RIT is time-sensitive and approach-avoidance conflicts may not be resolved immediately, it stands to reason that high BIS sensitivity may temporarily suspend cognitive task engagement. When additionally combined with high FFFS, this may effectively reduce the pool of generated reappraisal ideas.

Interestingly, there is specific evidence that high FFFS sensitivity according to the revised RST negatively predicts divergent thinking (Walker & Jackson, 2014), supporting the assumption that flight, freezing and active avoidance may reduce cognitive resources underlying higher order cognitive functions such as fluent idea generation for open-ended problems. Since reappraisal inventiveness in the RIT shares similar cognitive demands with traditional divergent thinking tasks (i.e., the generation of manifold alternative solutions to ill-defined problems; Fink et al., 2017; Weber et al., 2014), the BIS x FFFS interaction on reappraisal quantity may reflect a similar effect. Correspondingly, Jackson et al. (2014) found the flight dimension of the revised FFFS to be negatively related to executive functions like the inhibition of prepotent associations, which next to set-shifting and updating of working memory content is one of the key cognitions for successful reappraisal generation (e.g., Malooly et al., 2013; Rominger et al., 2018; Zaehringer et al., 2018). With the combination of high BIS x FFFS impeding cognitive control functions necessary for flexible situations re-interpretations, it is plausible that individuals may, at the expense of reappraisal ideas, resort to implementing other, less cognitively demanding regulation strategies like distraction or suppression in spite of task instructions or may be more prone to anger rumination (see Izadpanah et al., 2017). However, this interpretation is speculative pending further comprehensive analyses in larger samples.

In the present study, significant interaction effects of BIS/BAS/FFFS presented not only for total reappraisal inventiveness, but also for specific reappraisal tactics participants applied during the RIT. Here, note that while reappraisal inventiveness denotes an individuals’ capacity to come up with a large pool of distinguishable reappraisal ideas and thus indicates maximum performance (e.g., Papousek et al., 2017; Weber et al., 2014), individuals’ preference for using specific reappraisal tactics over others may be a more accurate reflection of how they spontaneously implement reappraisals in daily life. We observed an interaction of BIS and BAS on individuals’ preference for using positive re-interpretations, indicating that high BIS sensitivity may impede the construction of positive aspects in aversive scenarios when combined with low BAS sensitivity. While we initially hypothesized BAS sensitivity to be related to this reappraisal tactic based on shared links with reward processing and positive affect (Hundt et al., 2013; Shiota & Levenson, 2012), the additional involvement of BIS sensitivity further underlines the intricate interactions of motivational systems on shaping emotion regulation. Thus, these results offer additional support for the joint subsystems hypothesis in cognitive reappraisal generation (see Corr, 2002, 2013).

Previous studies hinted at the combination of high BIS-FFFS x low BAS being the most detrimental to adaptive emotion regulation, due to high BIS increasing negative emotional reactivity and low BAS reducing reward anticipation and emotion regulation efforts (e.g., Markarian et al., 2013, also see Pickett et al., 2012). High BIS and low BAS have also been associated with increased depressive, anxious, and stress-related symptoms (e.g., O’Connor et al., 2014; Harnett et al., 2013; Hundt et al., 2007). These findings align remarkably well with notions of positive re-interpretation being the most adaptive of cognitive reappraisal tactics. Accordingly, a positive reappraisal style is correlated with greater stress resilience (Kalisch et al., 2015), less negative emotional reactivity (Willroth & Hilimire, 2016), and less depression and anxiety (Everaert & Joormann, 2019). In a previous study with the RIT, individuals’ greater preference for utilizing positive re-interpretations for anger regulation was also directly correlated with lower chronic stress experience, whereas problem-oriented and de-emphasizing reappraisals were not (Perchtold et al., 2018). However, while deemed very effective, studies also suggest that positive re-interpretations are more cognitively demanding compared to other reappraisal tactics, since they require increased frontal control for switching from negative to not only neutral, but positive situational aspects (e.g., Rominger et al., 2018; Qi et al., 2017, also see Perchtold-Stefan et al., 2020). With high BIS prompting behavioral disengagement, and low BAS simultaneously reducing emotion regulation efforts (e.g., Markarian et al., 2013), their interaction may reduce individuals’ propensity to generate effective, yet effortful positive reappraisals.

Importantly, follow-up analyses revealed that the combined effect of high BIS and low BAS on positive re-interpretations only held for the BAS subdimensions of reward interest and impulsivity, but not reward reactivity and goal-drive persistence. While previous studies found positive correlations between general reward responsivity (according to the original RST), adaptive emotion regulation, and well-being (e.g., Markarian et al., 2013; Taubitz et al., 2015), our discrepant results for reward interest and reward reactivity are less straightforward. In the revised RST, reward interest denotes approach motivation in the face of potential reward independent of its actual presence, whereas reward reactivity refers to the experience of reward pleasure that reinforces subsequent behavioral approach (Krupić et al., 2016). Since the generation of positive reappraisals for anger-eliciting situations likely does not elicit immediate reward, but rather results in long-term benefits when repeatedly applied in critical contexts (e.g., Kalisch et al., 2015), this may explain why reward interest, but not reactivity interacted with the BIS on this reappraisal tactic. However, this assumption is speculative until further investigation.

Finally, high FFFS sensitivity negatively correlated with individuals’ preference for implementing problem-oriented reappraisals in the face of anger. In the present study, this effect was the only one independent from other motivational subsystems, indicating that individuals’ sensitivity to threat may directly impact their propensity for active problem solving and planning of remedial actions when confronted with anger-eliciting social situations. While not specifically concerned with reappraisal, previous studies demonstrated similar negative links between FFFS sensitivity and problem-focused coping as well planning abilities (Ivory & Kambouropoulos, 2012; see Vergara-Lopez et al., 2013 for a moderating effect of executive planning on the link between FFFS sensitivity and depression). Pending proper scoring methods, it will be interesting to see whether high FFFS sensitivity is linked to more passive, ruminative thinking during reappraisal generation, which is proposed to occur at the expense of active problem-oriented engagement in high avoidance individuals (see Manfredi et al., 2011).

Some limitations must be noted. First, the cross-sectional and correlational character of the present study discourages evaluations of causality regarding the associations between BIS/BAS/FFFS sensitivity and brain-based cognitive reappraisal capacity as well as preference for certain reappraisal tactics. While there is general consensus that individual differences in these three motivational subsystems causally predict emotion regulation (difficulties) and associated health outcomes (Izadpanah et al., 2016; Li et al., 2015, also see Azadi et al., 2020; Markarian et al., 2013), longitudinal investigations need to show that this direction of effects also hold for the concept of reappraisal inventiveness. Moreover, future studies are warranted to establish comprehensive models that not only scrutinize potential paths from BIS/BAS/FFFS sensitivity to reappraisal inventiveness, but also include direct and indirect paths to mental health and well-being. The sample was female only, necessitating replication in larger, mixed-sex samples. Resulting effect sizes were rather small, which may be concurrent with the notion that motivational systems are more strongly linked to maladaptive than adaptive emotion regulation (e.g., Tull et al., 2010; also see Markarian et al., 2013). However, also note that in the present study, individuals’ reappraisal inventiveness and preferred reappraisal tactics were assessed with a behavioral test and not by self-report, which may naturally reduce the formal size of expected correlations with reinforcement sensitivity questionnaires. Nonetheless, future RST models with reappraisal inventiveness would benefit from including individuals’ propensity for using certain maladaptive emotion regulation strategies as well (e.g., suppression, rumination,).

Despite these limitations, the present study provides the first comprehensive investigation of cognitive reappraisal generation within the revised RST framework, supporting links to both quantity and quality indicators of cognitive reappraisal operationalized as a brain-based capacity. Individuals high in BIS and FFFS generated a lower number of reappraisal ideas in the RIT, while individuals high in BIS and low in BAS showed a lower preference for implementing positive re-interpretations in the face of anger-eliciting events. Only higher FFFS sensitivity correlated with a lower preference for problem-oriented reappraisals independently of the other subsystems. Our findings largely substantiate joint effects of approach and avoidance motivation (Corr, 2002) and being based on separate measures for BIS and FFFS sensitivity according to the revised RST, emphasize their individual contribution as distinct, yet interacting motivational subsystems on cognitive reappraisal generation. Altogether, this study may inform future investigations on cognitive reappraisal as a crucial mechanism in the interplay of behavioral approach/avoidance and psychopathology.

## Data Availability

The data that support the findings of this study are available from the corresponding author upon reasonable request.

## Footnotes

1 Most studies on reinforcement sensitivity and emotion regulation used questionnaires based on the original RST (e.g., BIS/BAS Scales; Carver & White, 1994), and thus assessed combined BIS-FFFS sensitivity within the revised RST context (Corr, 2004; also see Tull et al., 2010). For this reason and since researchers are still combining the BIS and FFFS, we use the term “BIS-FFFS sensitivity” to discuss BIS findings of original RST literature.

2 Note that while Corr and Cooper (2016) suggested the Fight-Flight-Freeze system be retitled as “Flight-Freeze-Active Avoidance System (FFA)” for human studies, we use the FFFS terminology for better comparability with previous studies.
